# Cold Denaturation of Proteins in the Absence of Solvent: Implications for Protein Storage**

**DOI:** 10.1002/anie.202115047

**Published:** 2022-04-21

**Authors:** Emma L. Norgate, Rosie Upton, Kjetil Hansen, Bruno Bellina, C. Brookes, Argyris Politis, Perdita E. Barran

**Affiliations:** ^1^ Manchester Institute of Biotechnology University of Manchester Princess Street Manchester M1 7DN UK; ^2^ Department of Chemistry King's College London 7 Trinity Street London SE1 1DB UK; ^3^ Bristol-Myers Squibb Moreton Wirral CH46 1QW UK

**Keywords:** Cold Denaturation, Monoclonal Antibodies, Protein Folding, Structure–Activity Relationships, Variable-Temperature Ion Mobility

## Abstract

The effect of temperature on the stability of proteins is well explored above 298 K, but harder to track experimentally below 273 K. Variable‐temperature ion mobility mass spectrometry (VT IM‐MS) allows us to measure the structure of molecules at sub‐ambient temperatures. Here we monitor conformational changes that occur to two isotypes of monoclonal antibodies (mAbs) on cooling by measuring their collision cross sections (CCS) at discrete drift gas temperatures from 295 to 160 K. The CCS at 250 K is larger than predicted from collisional theory and experimental data at 295 K. This restructure is attributed to change in the strength of stabilizing intermolecular interactions. Below 250 K the CCS of the mAbs increases in line with prediction implying no rearrangement. Comparing data from isotypes suggest disulfide bridging influences thermal structural rearrangement. These findings indicate that in vacuo deep‐freezing minimizes denaturation and maintains the native fold and VT IM‐MS measurements at sub ambient temperatures provide new insights to the phenomenon of cold denaturation.

## Graphical abstract

Don't put your protein in the −20 °C freezer: a well‐known fact for biochemists and here it is demonstrated why this is right even in a vacuum. Variable‐temperature ion mobility allows measurement of the structure of isolated proteins at sub‐ambient temperatures. For three isotypes of monoclonal antibody, it is found that there is substantial structural rearrangement at 250 K (−20 °C), indicating a cold denaturation transition. At deep‐freezing temperatures (−80 °C) the structure is unaffected.

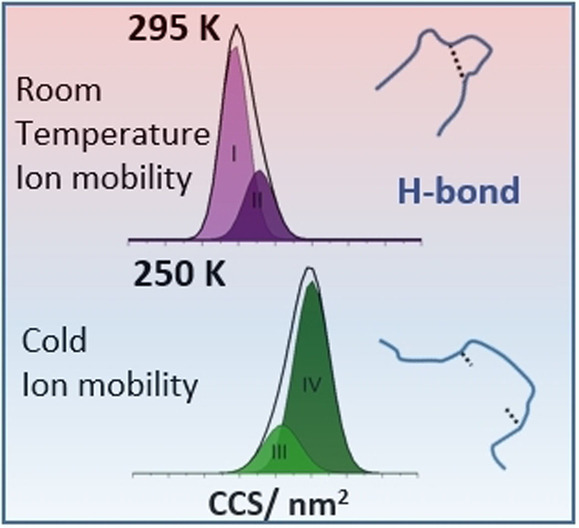

## Introduction

Cold denaturation is a phenomenon whereby proteins lose their tertiary and quaternary fold at low temperatures.^[1]^ In solution interactions between the solvent and the polar, hydrophilic groups on the exterior of the protein are in part responsible for maintenance of fold, as is the effect of polar solvent in driving hydrophobic groups to the protein interior. As the temperature of a polar solvent is lowered, solvent‐solvent interactions start to dominate and form strong networks that lead to the formation of ice in aqueous solutions. The effect of this is to weaken the influence of the solvent on the structure of the protein, which in turn will perturb the dependence of the fold in having hydrophilic groups on the exterior and hydrophobic on the interior. The free energy difference between the folded and unfolded state is very small and these effects can reverse the stability, causing the protein to denature.[^2^, ^3^] For large proteins, formation of ice in interstitial spaces may add to the perturbation of the tertiary fold, although the overall influence of lowering temperature on protein structure will be dependent on the time it takes to freeze the system.^[1]^ The effect of cold temperatures on the stability of proteins has relevance to biotechnology, for example in the development and/or manufacture of enzymes that work at low temperatures,[^4^, ^5^, ^6^, ^7^, ^8^] for biopharma, in the storage and transport of therapeutics[^9^, ^10^] and in the effects of a changing climate on agriculture and animals.[^11^, ^12^]

Very few experimental methods exist that can explore these effects, and yet, every protein biochemist is well aware that newly expressed proteins should be snap/flash frozen to −80 °C to preserve their functional fold, rather than placed in a −20 °C domestic freezer or fridge. By contrast, elevated temperature denaturation assays are a mainstay of biophysical evaluation of protein stability.[^13^, ^14^] The handful of studies on the effect of cold temperature on protein stability involve the use of a denaturant to destabilise the protein to a point where cold denaturation occurs at higher temperatures.^[15]^ Alternatively, the use of high‐pressure systems can be used to lower the freezing point of the water itself.^[16]^ However both these techniques inherently result in deviation of the protein from its native structure prior to the temperature being lowered, meaning the clarity between native and unfolded states is less distinct.^[17]^

**Figure 1:**
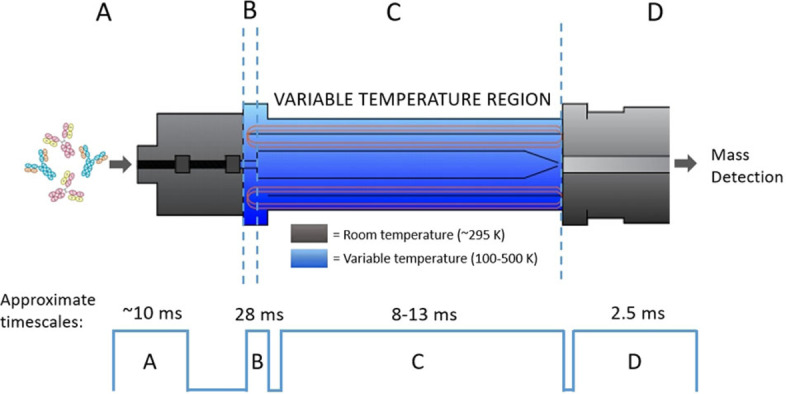
Schematic to show the main stages of VT‐IMS separation and the associated timescales. A) Protein sample enters instrument at room temperature via nESI. Transfer from liquid to gas phase takes approximately 10 ms.^[18]^ B) Protein ions are trapped forming spatially narrow “packets”. The ions are trapped for up to 28 ms in the ion buncher in the presence of buffer gas (helium), where they reach thermal equilibrium. C) Packets of protein ions enter the drift cell where they drift for 8–13 ms and separation based on the mobility of the ions occurs. D) Ions are transferred to a time of flight (ToF) mass analyser at room temperature and detected in the form of mass resolved arrival time distributions which can be converted to CCS distributions.

IM‐MS is uniquely positioned to study protein conformations and whilst most work is performed at ambient temperatures, there are instruments capable of measurements from 80 K to 600 K including the one used in this study (Figure 1).[^19^, ^20^, ^21^, ^22^] The collision cross section measured in all IM‐MS experiments is temperature dependent^[23]^ and VT IM‐MS instruments are able to measure both the effect of temperature on the structure of protein ions as well as the effect of temperature on CCS. Due to the increased influence of the long‐range interactions at low temperatures, the CCS of a rigid ion will increase as the temperature is lowered. The resolution of ion mobility increases with (√T)^−1^ which means this approach could resolve conformers not possible at ambient temperatures. Monoclonal antibodies (mAbs) have inherent flexibility around and remote from the pivotal hinge region and therefore are good target molecules to be examined by VT IM‐MS. They exhibit broad CCS distributions consisting of several closely related conformational families.^[24]^ IM‐MS has shown that it is possible to differentiate the conformational landscapes adopted by IgG subclasses/ isoforms as well as determine the impact of glycosylation on stability.[^24^, ^25^, ^26^, ^27^] VT IM‐MS experiments on mAbs over an elevated temperature range (300–550 K) showed how these proteins unfold in vacuo.^[28]^ Here we explore thermally induced conformational change below room temperature.

## Results and Discussion

VT IM‐MS measurements of IgG1 (Herceptin), IgG2 (GMP3) and NIST mAb samples were performed on a home‐built IM‐MS instrument as previously described (Figure 1).^[29]^ In brief, ions are generated via nanoESI (source temperature 80 °C, capillary voltage ≈1.0–1.4 kV), and are guided by two ion tunnels towards an ion buncher which is located within the high pressure region helium (≈2 Torr) where they are accumulated prior to entering the 50.5 cm drift region (Figure 1). The drift tube comprises a double jacket design; an external stainless‐steel chamber encompassing an internal insulating tube housing the electrode stack and buffer gas. Copper tubing arranged in a longitudinal direction is situated between the two jackets. The copper tubing extends beyond the drift region and is connected to the house N_2_ gas supply which is passed through liquid nitrogen to generate a coolant. Fine tuning of the N_2_ flow rate determines the flow of coolant through the tubing surrounding the drift cell and therefore allows control of the drift gas temperature to ±1 K (see Supporting Information S1). Arrival time distributions were acquired over a range of drift voltages (290–220 V) before being converted to CCS distributions as described previously.^[24]^

**Figure 2:**
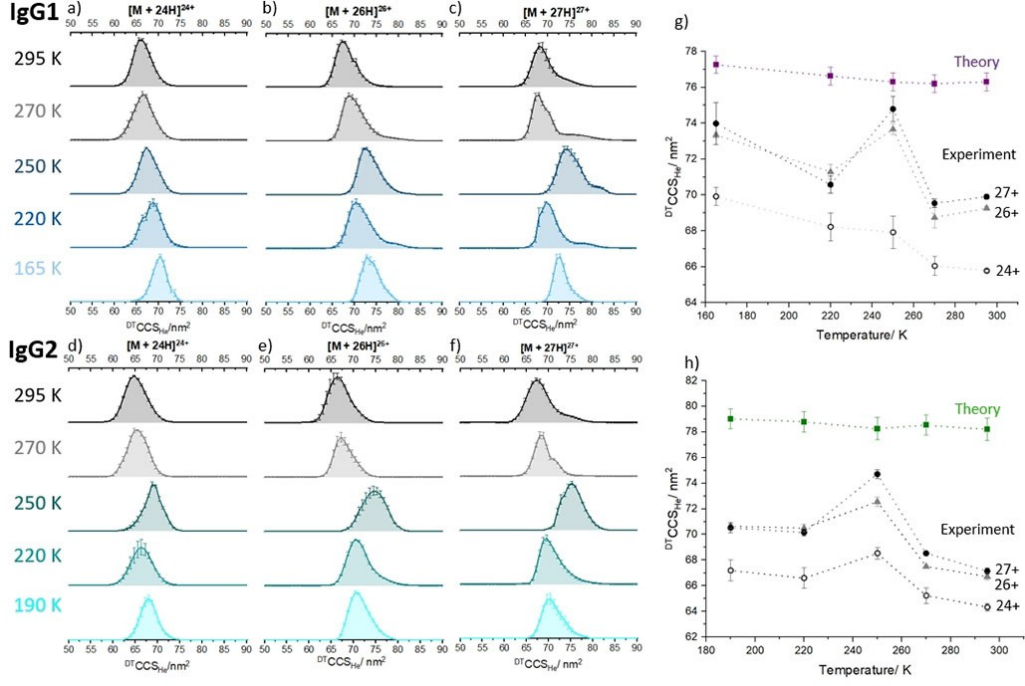
Collision cross section distributions (^DT^CCSD_He_) for a) 24+, b) 26+ and c) 27+ charge state ions of IgG1 at five temperatures from 295 to 165 K; and d) 24+, e) 26+ and f) 27+ charge state ions of IgG2 at five temperatures from 295 to 190 K . Error bars correspond to the standard deviation from three, 3 minute long acquisitions. The 25+ charge state for both antibodies is presented in Supporting Information Figure S6. Projection Superposition Approximation (PSA)[^30^, ^31^, ^32^, ^33^] Calculated CCS values (^CALC^CCS_PSA_) are shown for IgG1 in g) (purple squares) and for IgG2 in h) (green squares), with experimental ^DT^CCS_He_ values for [*M*+24 H]^24+^, [*M*+26 H]^26+^ and [*M*+27 H]^27+^ (solid black circles, grey triangles and hollow black circles, respectively).

VT IM‐MS data was obtained for three mAbs over the temperature range 295–165 K. Initial room temperature CCS distributions are in line with previous measurements.[^28^, ^34^] We observe a more restricted CCS distribution for IgG2, demonstrating lower flexibility compared with IgG1 and NIST mAb. Further details and discussion of room temperature measurements for IgG1, IgG2 and NIST mAb (Figures S2 and S3) is contained in the Supporting Information. Figure 2 depicts the collision cross section data for selected charge states of IgG1 and IgG2, in the forms of ^DT^CCS_He_ distributions (Figure 2a–f) and apex values (Figure 2g, h). For all ions the ^DT^CCS_He_ increases as a function of reduced temperature, in line with theory.^[35]^ The magnitude of the CCS for each charge state at all temperatures is different, with the higher charge states corresponding to larger forms of the protein (Figure S2). This can be explained in terms of the charge imparted by the nESI process sampling different conformational distributions.^[36]^ For IgG1, comparing measurements taken at 295 K with 165 K, we observe an increase in ^DT^CCS_He_ of ≈6 % for all charge states. For IgG2 we observe similar overall increases for each charge state of ≈5 % from 295 to 190 K. It is also notable that the CCS distribution at 165 K is substantially narrower, indicating higher resolution at this cryogenic temperature.

Whilst the overall effect of lowered temperature is to increase the CCS, at 250 K there is an interesting exception to this predicted trend, particularly for the higher charge states. The theory for the transport of ions in gases does not permit any prediction to be made that there should be significant differences in the ion‐neutral interaction potentials for each charge state at a given temperature,^[37]^ although the resolution will increase as the temperature is lowered (*R*_max_ α 1/$\sqrt{T})$ .^[38]^ Here at 250 K we observe a larger than predicted increase in ^DT^CCS_He_, and significant broadening of the distribution of conformers. Notably the effects are more pronounced for higher charge states, with the ^DT^CCS_He_ of [IgG1+24H]^24+^ increasing by 4 % from 295 K to 250 K, whereas for [IgG1+27H]^27+^ the increase is 7 %. For IgG2 the effect is even more apparent, Δ ^DT^CCS_He_ is +6 % for [IgG2+24H]^24+^ and +11 % for [IgG2+27H]^27+^. It has been observed in previous work that higher charge states unfold more readily due to a lower energy barrier between folded and unfolded states,[^39^, ^40^] and here we are likely observing a similar effect when comparing higher and lower charge states.

These observations can be explained by the existence of conformational ensembles which are more variable at higher charge states and differently affected by the low temperatures. Some of the dehydrated conformers that populate the higher charge states yield ^DT^CCS_He_ values that are much larger than predicted. There is a significant increase in the full width half height maximum (FWHM) of the [IGg1+27H]^27+ DT^CCSD_He_ at 250 K. These observations indicate restructuring of the conformers to more extended states at 250 K, which is in good agreement with previous predictions of cold denaturation for mAbs in solution.^[41]^

For the NIST mAb, we also observed comparable increases in ^DT^CCS_He_ with decreasing temperature (Figure S5). Higher charge states of this mAb possess a significant population of more extended conformers at 295 K, Figure S3a) and the relative increase in ^DT^CCSD_He_ width at 250 K is a lot more subtle, cf. that found for IgG1 and IgG2. This implies that when a dehydrated mAb occupies a larger conformational range at room temperature, it is less prone to cold denaturation and that the effects that we observe are representative of the desolvated structure, which is in turn reliant on the non‐covalent interactions dictated by the primary sequence.

Before we start to consider why this conformational effect is indeed dependent on the dehydrated tertiary fold we note that for each mAb their mass spectra (Figure S4) are unchanged as a function of temperature. We also observe only minor differences in the associated salt retention, based on the width of the MS signals; thereby discounting adduct effects upon the ^DT^CCS_He_ distributions. To quantify the extent of the increase in CCS at 250 K we compare temperature dependant experimental ^DT^CCS_He_ values with theoretical Projection Superposition Approximation (PSA) calculations (^CALC^CCS_He_) on dehydrated mAbs.^[42]^ CCS calculations were performed using PSA CCS calculation webserver.[^30^, ^31^, ^32^, ^33^] The PSA values are shown in Figure 2g, h and whilst they indeed show the expected increase in CCS as the drift gas temperature is lowered, the difference between 295 and 250 K is minimal, far less than observed experimentally. We also note here that the magnitude of the increase between 295 and 165 K is lower in the theoretical model than experiment.

**Figure 3:**
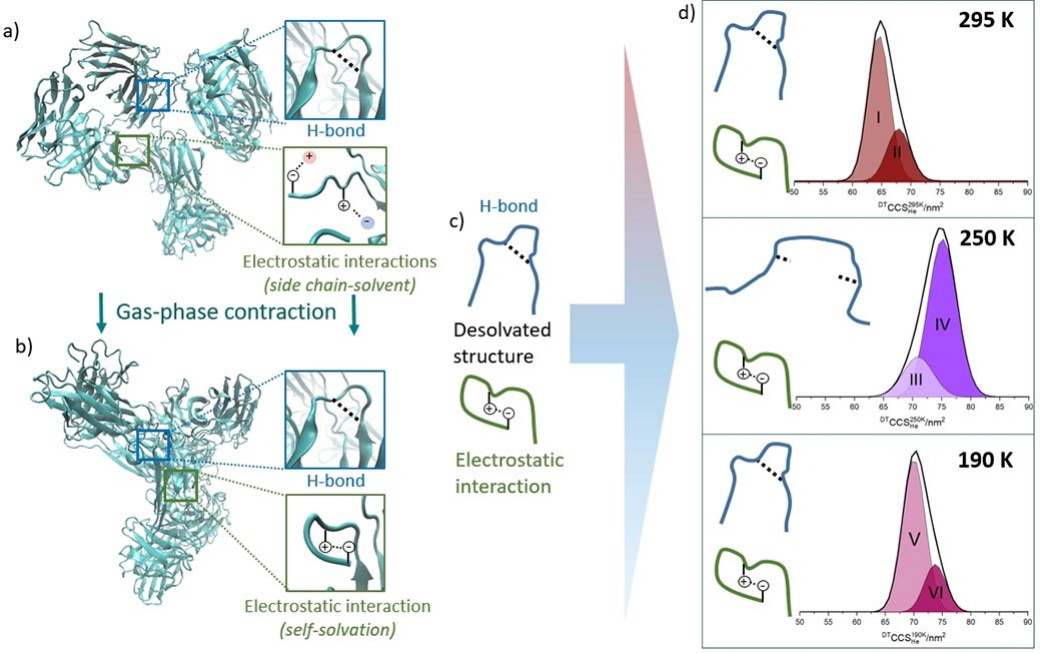
Schematic representation of structural changes that occur upon dehydration and ion mobility measurements at low temperature for IgG2. Ribbon diagrams show a) the starting solution structure and b) the energy minimised gas phase structure^[42]^ of IgG2. Illustrative examples of hydrogen bonds (blue boxes) and electrostatic interactions (green boxes) are shown for solution conformers (a) and the dehydrated form (b). The entire conformational ensemble is then placed in the drift cell at three different temperatures and the effect on the dominant non‐covalent interactions (c) is shown in (d) along with the summed ^DT^CCS_He_ distributions for IgG2 at 295 K, 250 K and 190 K fitted with Gaussian functions for two conformers at each temperature.

Summing the collision cross section distributions for each charge state allows the magnitude of this structural deformation at 250 K on the entire conformational ensemble to be visualised (Figure 3d and S7). IgG1 and IgG2, at 295 K each present two dominant conformational families. At the lowest temperature sampled (165 K) the relative population of each conformer is highly similar to that at 295 K (Figure 3d and S7), albeit with an increase predicted by theory (although the magnitude appears greater than predicted by the PSA model (Figure 2g and h).[^30^, ^31^, ^32^, ^33^] This indicates that the dehydrated structures do not alter substantially when plunged into the drift cell at near cryogenic temperatures, and that we need to revise the theoretical understanding of the effect of temperature on CCS. This means that ion mobility data taken at different temperatures may act as a reference set for cryo‐EM structures as well as provide insights for double electron‐electron resonance (DEER) measurements. In fact, cryo‐EM has some limitations in examining antibodies due to their relatively low molecular weight and highly flexible structure,^[43]^ demonstrating that VT‐IMS can provide unique structural information for antibodies at cryogenic temperatures. By contrast, the CCS distribution at 250 K is markedly different and much larger in magnitude, we have also noted this for lysozyme.[^44^, ^45^] For IgG2 the percentage occupancy of conformer (II) is substantially greater at 250 K. This is also the case with IgG1 although not as marked. (Figure 3, and Figure S7g, h) The increase in apex ^DT^CCS_He_ is 10 % from peak I to III and 11 % from peak II to IV for the IgG2 sample. This suggests that for IgG2 at 250 K, a greater proportion of the ions restructure to occupy more extended conformations than with IgG1.

The VT‐IMS data presented here show clear evidence of cold denaturation of IgG1 and IgG2 antibodies at 250 K implying a denaturing transition that occurs at this temperature. These gas phase measurements agree with studies performed in solution,^[41]^ which raises the question is cold denaturation a phenomenon that occurs independent of solvent?

Cold denaturation is a well‐established characteristic of eukaryotic proteins.[^3^, ^46^, ^47^] A consensus view is that cold denaturation occurs as water starts to freeze, which in turn lessens the hydrophilic stabilisation of the protein fold imparted by interactions between charged residues on the surface of the protein and bulk solvent. This is attributed to the lengthening and stiffening of water‐water H‐bonding, which interrupts the attractive interaction of the protein hydrophilic sites with bulk solvent.[^46^, ^48^, ^49^] The surprising aspect of the data presented here is that we observe cold denaturation in an environment deplete of bulk solvent, and hence we need to rationalise how a self‐solvated dehydrated macromolecule can also undergo cold denaturation.

Figure 3 illustrates what we speculate is the effect on stabilising non‐covalent interactions of the transition of a mAb from solution to gas phase (Figure 3a, b), and subsequently into a drift cell at a given temperature (Figure 3c). During the nESI process, there are progressively fewer solvent molecules with which the protein side‐chains can form stabilising interactions.[^18^, ^50^, ^51^] One effect of this is that oppositely charged side‐chains, in the absence of a buffer counter‐ion, may form stabilising intermolecular electrostatic interactions, causing an overall structural contraction *cf* the solvated state.^[52]^ The H‐bonds that are present in secondary structural elements within the protein may also contract a little on desolvation also due to the removal of any solvent screening.

We hypothesise that, similarly to the freezing of water, lowering the temperature induces more rigid, extended hydrogen bonding (Figure 3d). When this effect is coupled to residual protein dynamics, at *T*=250 K, H‐bonds may extend so much that they are no longer stabilising and in the absence of any solvent, this results in regions such as beta sheets, becoming detached from one another.^[46]^ Coulombic theory provides no rationale for electrostatic bonds being altered by changes in temperature, but the tightening on dehydration may provide stiffer regions which are prone to decouple as the H‐bonds extend. The fact we observe these effects most strongly at 250 K suggests that the residual dynamics allow this to occur on the timescale at which the protein experiences the cooling. At lower temperatures the residual dynamics must be dampened sufficiently fast to prevent significant restructuring, hence we observe no change in the relative population of conformers (Figures 2, 3d and S7).

It is well established in literature that cold denaturation occurs due to the change in the solvated environment, water effectively becomes more hydrophobic as it cools, with fewer H‐bonds to the protein at lower *T*, and furthering the solvation of hydrophobic regions, which in turn drives the unfolding of the protein.[^49^, ^50^] Our experiment indicates that the phenomenon of cold denaturation can also occur due to inherent interactions in the tertiary and quaternary structure, which has implications for the effect of cold temperature on dehydrated proteins in cold environments, and may explain why so called cold adaptive proteins are often disordered in solution with sequences that adapt to more rigid structures in cold dry environments.

Further insights are gained by comparing the behaviour of the IgG1 with the IgG2 mAbs. The main structural difference between these two sub‐families is found in the hinge; IgG2 possesses more disulfide bridges than IgG1 and consequently lower flexibility than IgG1.^[42]^ For IgG1, the flexibility of the native structure means that a wide range of conformations are sampled in the gas phase (Figure S7b).[^24^, ^42^] Conformers that present lower charge states of IgG1 are largely unaffected at 250 K (Figures 2a), but those in higher charge states show an increase in CCS to occupy more extended structures (Figures 2c). IgG2 behaves differently. The conformers present over all the charge states are affected by the cold and restructure to more extended forms (Figures S7d–f and h). We surmise that the low flexibility of this antibody means that conformers ‘snap’ away from the dehydrated folded state to more extended forms at 250 K. This is not observed for IgG1 where we suggest the higher intrinsic dynamics mean that the non‐covalent networks remain more able to preserve the dehydrated forms.

## Conclusion

In conclusion, this study demonstrates how VT IM‐MS data can provide unique insights into cold denaturation at a molecular level. For IgG1 and IgG2, comparing measurements taken with the drift gas at room temperature to measurements at 250 K, we observe larger‐than‐expected CCS values and a greater occupancy of more extended conformers, as well as broadening of the conformational distribution: a phenomenon our earlier work on monomeric proteins has hinted at.^[45]^ At temperatures below 250 K, the occupancy of conformers is very similar to that at room temperature, although the distributions are narrower suggesting that the protein ions are “snap frozen” and no structural rearrangement can occur. All these factors act as clear evidence of cold denaturation of IgG1 and IgG2 in the absence of solvent at 250 K (−20 °C).

This evidence for cold denaturation in the absence of solvent implies that the factors governing it are intrinsic to the fold. The large positive heat capacity of unfolding drives cold denaturation and whilst this has previously been attributed to solvent solute effects, it is clear that this can be driven by changes to the strength of non‐covalent interactions. Our VT‐IM‐MS data implies that the primary sequence and consequent fold of a given protein can dictate its cold denaturation behaviour. It is remarkable that the cold denaturation in vacuo is dominant at 250 K which is what has been predicted in the solvated state. Future work will consider how this approach can inform our understanding of living systems that adapt to live in cold environments as well as provide reference structures for cryo‐EM.

## Conflict of interest

The authors declare no conflict of interest.

1

## Supporting information

As a service to our authors and readers, this journal provides supporting information supplied by the authors. Such materials are peer reviewed and may be re‐organized for online delivery, but are not copy‐edited or typeset. Technical support issues arising from supporting information (other than missing files) should be addressed to the authors.

Supporting Information

## Data Availability

The data that support the findings of this study are available from the corresponding author upon reasonable request.
