# Supplementary material for: Cold Denaturation of Proteins in the Absence of Solvent: Implications for Protein Storage
Source: Angew Chem Int Ed Engl. 2022 Apr 21;61(25):e202115047. doi: 10.1002/anie.202115047 (PMC9325448; doi:10.1002/anie.202115047)
Supplement: Supplementary file 1 — Supporting Information [file ANIE-61-0-s001.pdf]

Angewandte Chemie
Eine Zeitschrift der Gesellschaft Deutscher Chemiker

Supporting Information

Cold Denaturation of Proteins in the Absence of Solvent: Implications for Protein Storage

*E. L. Norgate, R. Upton, K. Hansen, B. Bellina, C. Brookes, A. Politis, P. E. Barran**

Supporting Information

Materials

Herceptin was a gift from UCB Pharma and the NIST monoclonal antibody reference material 8671 was purchased from NIST.[51] GMP3 IgG2 antibody was a gift from Allergan Biologics. Ammonium acetate was purchased from Fisher Scientific. Purified water was produced in-house using a Milli-Q Advantage A10 system. All samples were prepared at 1 mg/ml (~5 µM) in 100 mM ammonium acetate, pH 6.80 and desalted using micro Bio-Spin 6 columns (Bio-Rad) prior to analysis.

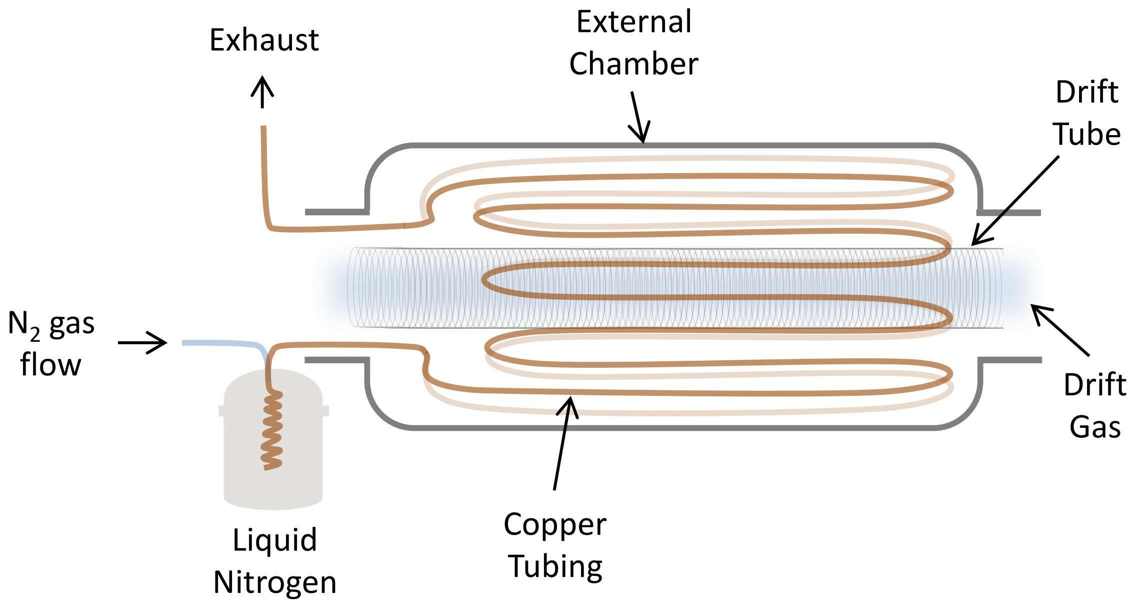

Figure S1. *A schematic of the cooling system for the VT-IM-MS drift tube for cold temperature IM experiments – not to scale.*

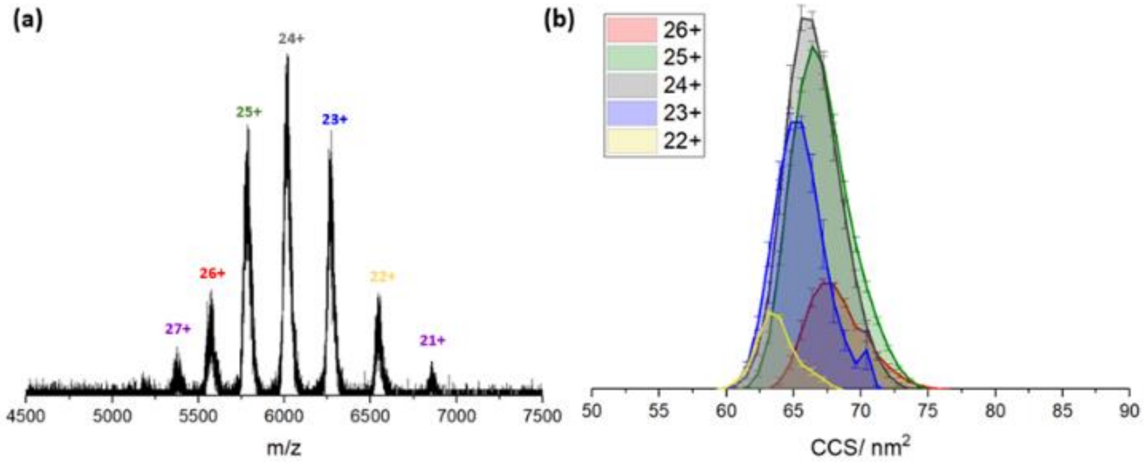

Figure S2. *(a) Mass spectrum of Herceptin showing charge states ranging from 27+ to 21+. (b) Collision cross section distributions ($^{DT}CCSD_{He}$) for the most intense charge states of Herceptin following nano-ESI. Data shown was acquired at ~295 K and at equivalent drift voltages. Charge states have been normalised to the mass spectral intensity. Error bars represent standard deviation from three replicates.*

The mass spectra for Herceptin (Measured mass: 148836 Da; Theoretical mass: 145071.9 Da) (previously reported[1], Figure S1a) present narrow charge state distributions diagnostic of a native-fold; with the charge state envelopes ranging from 21+ to 27+ and both centred at 24+. The experimentally measured mass is higher than the theoretical mass due to the presence of N-linked glycosylation (~ 1400 – 1700 Da per heavy chain) as well as residual salt and solvent molecules following nESI. The remaining mass increase would equate to the addition of around 20 to 55 water molecules or 6 to 18 sodium chloride molecules. Figure S1b presents the room temperature $^{DT}CCSD_{He}$ for the five most populated charge states of Herceptin. Each charge state represents a snapshot of the conformational ensemble of the mAb, with some overlap in the conformational properties of different charge states, as shown previously.[2]

For all three mAbs we see an increase in the $^{DT}CCS_{He}$ as the overall charge on the mAb increases. Although it has been demonstrated for some proteins that Coulombic repulsions do affect high charge states, causing unfolding and resulting in an increase in $^{DT}CCS_{He}$,[3] the $^{DT}CCS_{He}$ increases that we observe are more likely attributed to the inherent conformational heterogeneity of the mAbs. The maximum number of surface charges (z_{max}) that a quasi-spherical protein can accommodate before their proximity results in Coulombic repulsion has been theorised;[4] the charge states we report here however, are below the predicted z_{max} limit.[5]

Similar MS and IM-MS data has been obtained for our IgG2 GMP3 sample (SI Figure S4c and S3b respectively), and for comparison MS and IM-MS data of a reference mAb standard (NIST; delivered and stored at -80 °C) was acquired (Figure S4b and Figure S3a, respectively). In both samples a narrow, native charge distribution is presented (21+ to 27+; centred at 25+ for NIST mAb and at 24+ for IgG2 GMP3). A restricted $^{DT}CCSD_{He}$ was observed for both, with IgG2 being significantly more restricted than NIST mAb (NIST mAb $^{DT}CCSD_{He}$ overall ~25 nm^2 and FWHM ~10 nm^2; IgG2 GMP3 $^{DT}CCSD_{He}$ overall ~15 nm^2 and FWHM ~6 nm^2) due to the lower flexibility of IgG2 compared with IgG1 and NIST mAb.[6] For the NIST mAb we measured a $^{DT}CCS_{He}$ of 64.7 nm^2 for the $[M+22H]^{22+}$ ions which increased to 69.9

nm^2 for the $[M+26H]^{26+}$ ions; our results are in-line with previously reported NIST mAb $^{DT}CCS_{He}$ values (67.6 – 68.9 nm^2).[7]

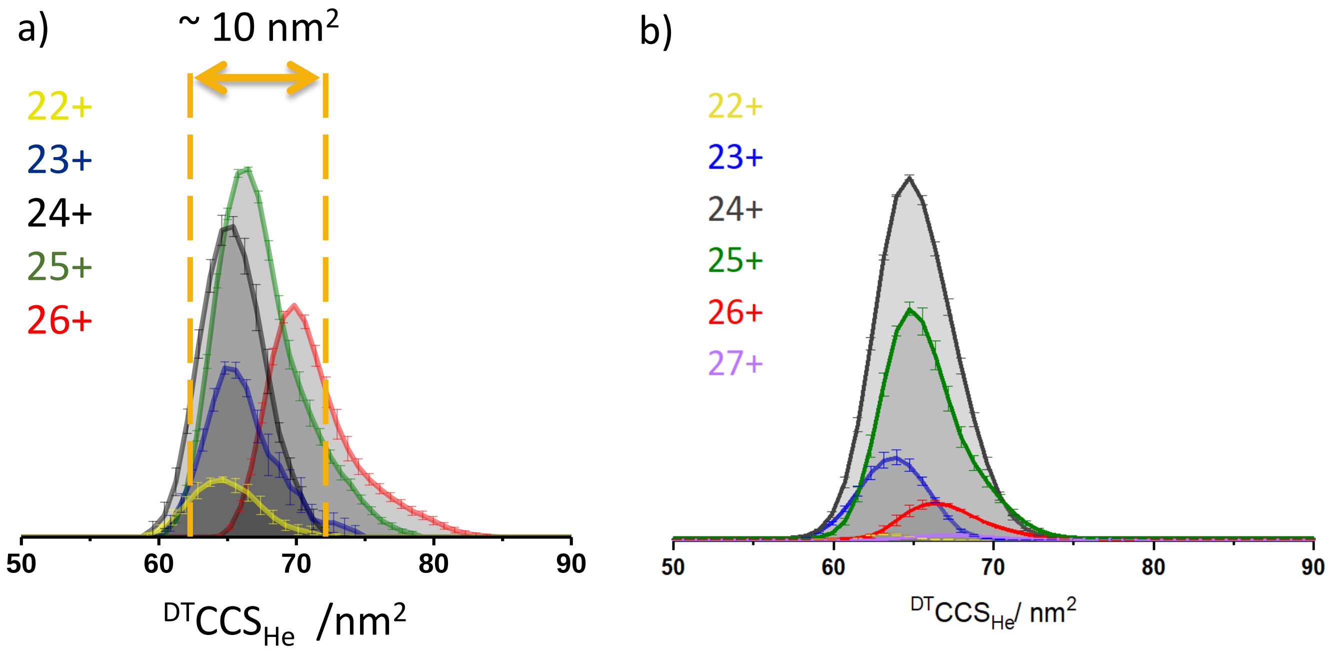

Figure S3. *Collision Cross Section Distributions ($^{DT}CCSD_{He}$) for the most intense charge states of (a) reference standard NIST mAb and (b) IgG2 GMP3 following nESI (5 µM in 100 mM ammonium acetate). Data shown was acquired at ~295 K and a drift voltage of 260 V. Error bars correspond to the standard deviation from three replicates. Data has been normalised to the mass spectral intensity (mass spectrum provided in Figure b).*

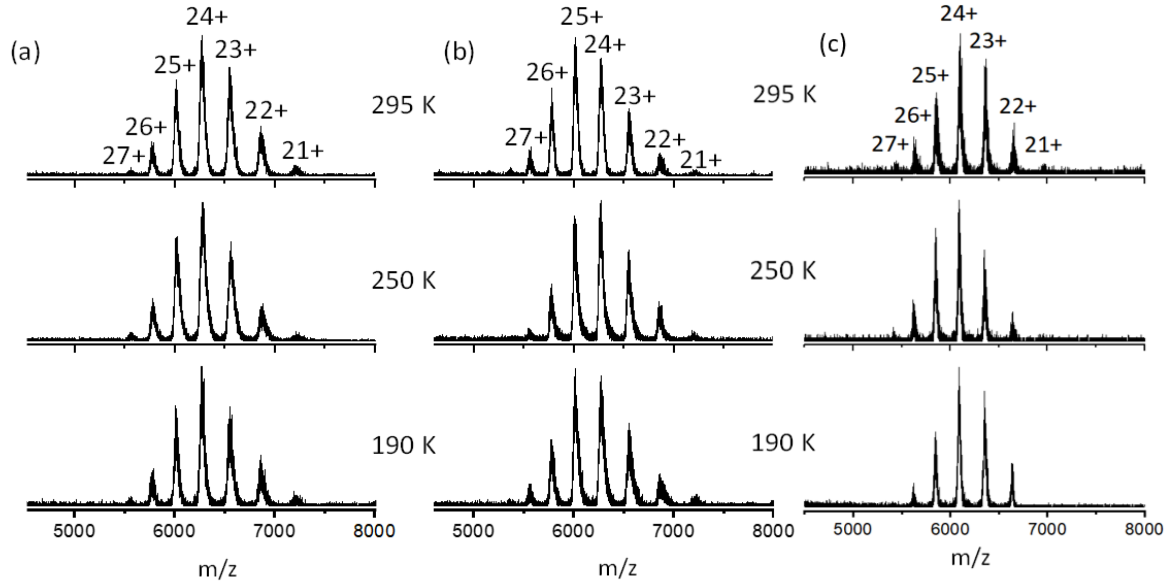

Figure S4. *Mass spectra for (a) IgG1 Herceptin, (b) the NIST mAb and (c) IgG2 GMP3 at three different temperatures; 295, 250 and 190 K. Any differences in the intensity ratio between charge states over 295 – 190 K reflects the variation in day-day fine tuning of the nESI source. Theoretical monoisotopic masses for Herceptin and the NIST mAb based upon their amino acid sequences are 145071.9 Da and 145342.28 Da, respectively.*

Experimentally measured masses at 295 K were determined as 148836 ± 123 Da and 148880 ± 116 Da, respectively. These differences in mass can be attributed to the presence of N-linked glycosylation (~ 1400 – 1700 Da per heavy chain) as well as residual salt and solvent molecules following nESI. Measured masses at 250 K and 190 K cf. 295 K increase by ~ 0.10 % and ~0.03 % for Herceptin, respectively and by ~0.04 % and ~0.06 % for the NIST mAb, respectively.

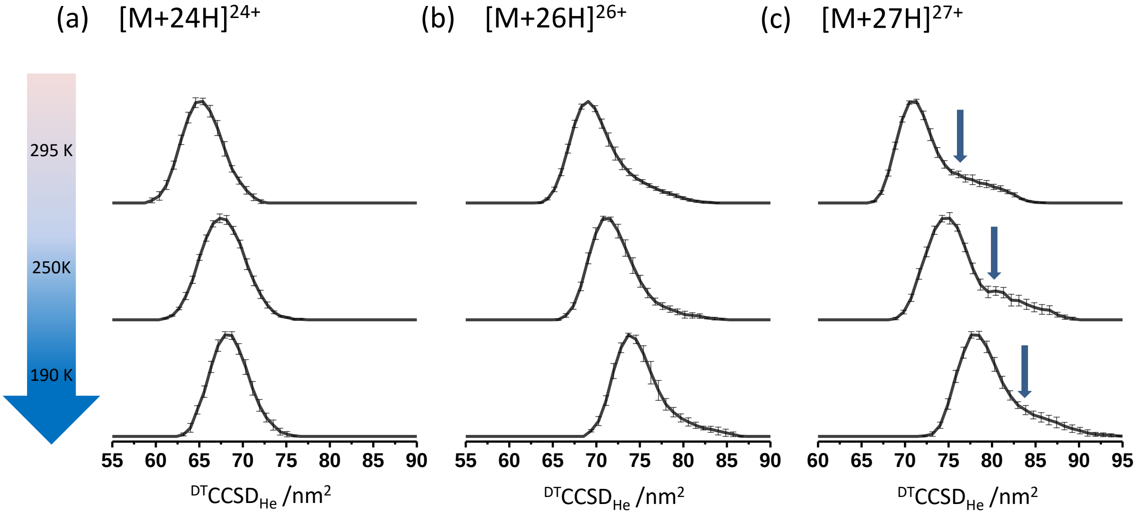

Figure S5. *Collision cross section distributions ($^{DT}CCSD_{He}$) for (a) $[M+24H]^{24+}$, (b) $[M+26H]^{26+}$ and (c) $[M+27H]^{27+}$ ions of the NIST mAb at three temperatures; 295, 250 and 190 K. Error bars correspond to the standard deviation from six, 1 minute long acquisitions. Blue arrows indicate the position of an extended conformational family.*

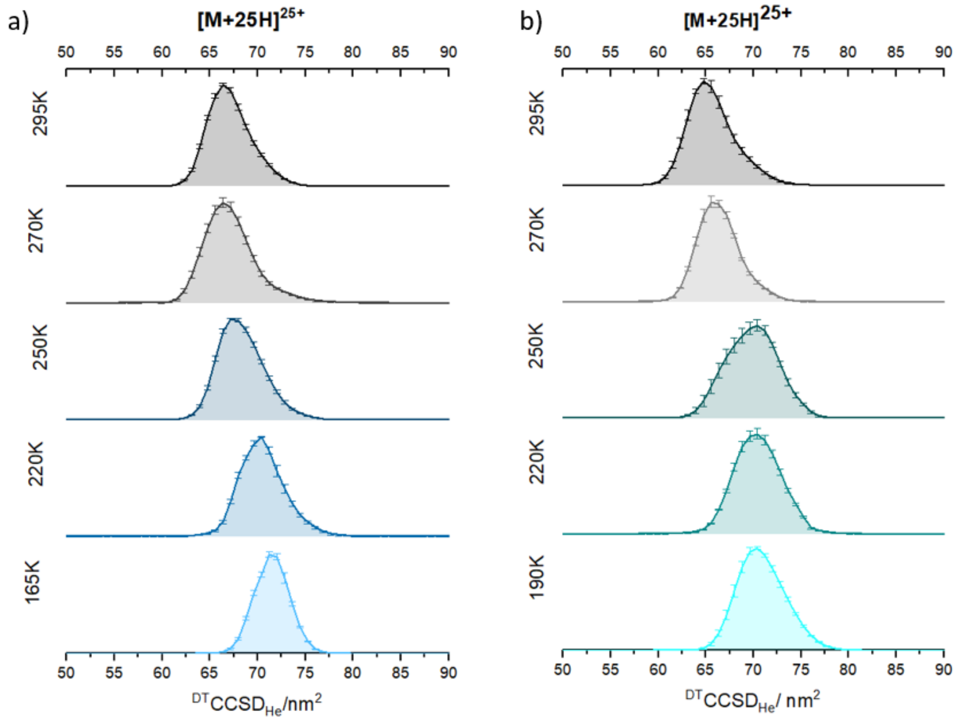

Figure S6. *Collision cross section distributions ($^{DT}CCSD_{He}$) for $[M+25H]^{25+}$ charge state of (a) Herceptin and (b) GMP3 at temperature between 295 and 165 K.*

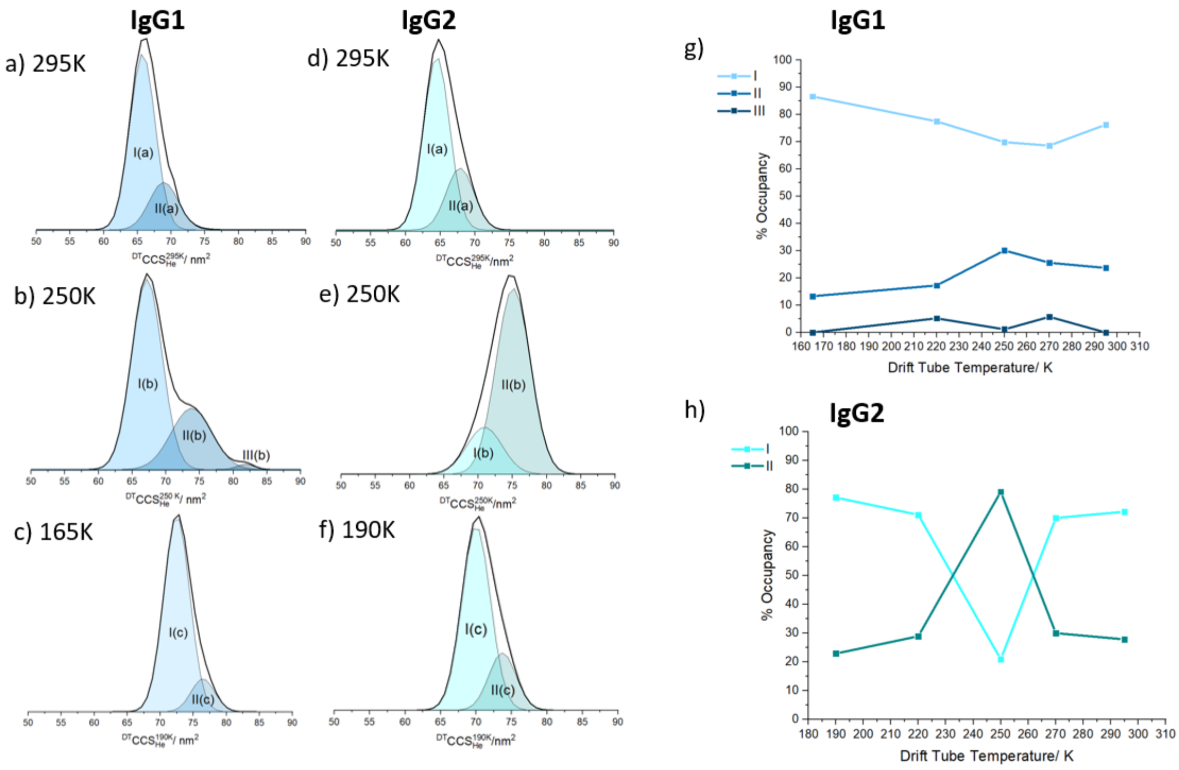

Figure S7. *Overall collision cross section distributions ($^{DT}CCSD_{He}^{T}$) for Herceptin with the drift tube at temperature a) 295 K, b) 250 K and c) 165 K, and for GMP3 at temperature d) 295 K, e) 250 K and f) 190 K. All charge states are scaled to their MS intensity and summed together to yield the global $^{DT}CCSD_{He}^{T}$. $^{DT}CCSD_{He}$ peaks are fitted with Gaussian peaks which correlate to conformers I (light blue), II (blue) and III (dark blue) for Herceptin (a-c), and conformers I (light turquoise) and II (dark turquoise) for GMP3 (d-f), for each temperature. Conformer I is the more compact, and II and III are more extended. The % occupancies of conformers I, II and III for Herceptin at temperatures 295K to 165K are shown in part g) and for conformers I and II of GMP3 at temperatures 295 K to 190 K in part h).*

Herceptin (IgG1) displays a significant widening of $^{DT}CCS_{He}$ distribution at 250 K. At this temperature peak I increases in $^{DT}CCS_{He}$ by 2%, and peak II by 7% w.r.t 295 K (Figure S6 parts a) and b)). We also observe a third, even more extended conformer III at 250 K for Herceptin. The base width of the global $^{DT}CCSD_{He}$ for Herceptin increases from 18 nm^2 at 295 K to 26 nm^2 at 250 K: a 43% increase. At 250 K, GMP3 (IgG2) displays a narrower range of conformational families compared to Herceptin, with a $^{DT}CCSD_{He}$ base width increase of 34% from 295 K to 250 K (Figure S6). Interestingly, for GMP3 the percentage occupancy of unfolded conformer (II) increases significantly at 250 K, to a much greater extent than Herceptin, as shown in Figure 3 parts g) and h). The percentage increase in $^{DT}CCS_{He}$ is 10% for peak I and 11% for peak II for the GMP3 sample.This suggests that for GMP3 (IgG2), a much greater proportion of the ions unfold to occupy more extended conformations.

Generating initial models and energy minimisation of IgG1 and IgG2

All generation of initial models and energy mimisation was performed by K. Hansen *et al.,* Kings College London. Full information of model generationa and energy minimisation is detailed by K. Hansen elsewhere.[6] All modelling was performed using MODELLER.[8] In short, for IgG1 1HZH(human) and 1IGY (mouse) templates from the PDB were used. For IgG2, 1IGT (mouse; whole molecule), 4L4J

(human; Fc) and 2QSC (human; Fabs) structures were used. Two additional disulphide bonds were inserted into the hinge region for IgG2 to obtain an accurate model of human IgG2.

Both IgG1 and IgG2 structures were energy minimised using GROMACS 5.1.3.[9] IgG models were pre-charged using a locolised charging model. Simulations were performed using the OPLS forcefield. Further details of energy minimisation and simulations are detailed elsewhere[6].

CCS calculations

Theoretical CCS calculations were performed using Projection Superposition Approximation (PSA) webserver. The output values are tabulated in Table 1.

	**Herceptin IgG1**		**GMP3**	
Temperature	CCS/ nm^2	Error	CCS/ nm^2	Error
295 K	76.27	0.43	78.20	0.48
270 K	76.20	0.43	78.53	0.48
250 K	76.30	0.44	78.25	0.48
220 K	76.63	0.44	78.79	0.49
190 K	76.89	0.44	79.02	0.49
165 K	77.26	0.44	79.38	0.49

Table 1. *PSA output values for Herceptin and GMP3*

Job.config file values are shown:

```
psa.general.buffergas=helium
psa.general.temperature=295, 270, 250, 220, 190, 165
psa.projection.accuracy=0.01
psa.projection.integration.accuracy=0.009
psa.shape.accuracy=0.1
psa.shape.maxiter=5000
psa.shape.meshfactor=1
```

References

1 K. J. Pacholarz, M. Porrini, R. A. Garlish, R. J. Burnley, R. J. Taylor, A. J. Henry and P. E. Barran, *Angew. Chemie - Int. Ed.*, 2014, **53**, 7765–7769.

2 C. Pritchard, G. O. Connor and A. E. Ashcroft, *Anal. Chem.*, 2013, **85**, 7205–7212.

3 Z. Hall, A. Politis, M. F. Bush, L. J. Smith and C. V. Robinson, *J. Am. Chem. Soc.*, 2012, **134**, 3429–3438.

4 J. Fernandez De La Mora, *Anal. Chim. Acta*, 2000, **406**, 93–104.

5 R. Beveridge, S. Covill, K. J. Pacholarz, J. M. D. Kalapothakis, C. E. Macphee and P. E. Barran, *Anal. Chem.*, 2014, **86**, 10979–10991.

6 K. Hansen, A. M. Lau, K. Giles, J. M. McDonnell, W. B. Struwe, B. J. Sutton and A. Politis, *Angew. Chemie - Int. Ed.*, 2018, **57**, 17194–17199.

7 I. D. G. Campuzano, C. Larriba, D. Bagal and P. D. Schnier, in *State-of-the-Art and Emerging Technologies for Therapeutic Monoclonal Antibody Characterization Volume 3. Defining the Next Generation of Analytical and Biophysical Techniques*, American Chemical Society, 2015, vol. 1202, pp. 4–75.

8 A. Fiser, R. K. Do and A. Sali, *Protein Sci.*, 2000, **9**, 1753–1773.

9 H. J. C. Berendsen, D. Vanderspool and R. Vandrunen, *Comput. Phys. Commun.*, 1995, **91**, 42–56.
